# Association between atherogenic lipids and GnRH agonists for prostate cancer in men with T2DM: a nationwide, population-based cohort study in Sweden

**DOI:** 10.1038/s41416-022-02091-z

**Published:** 2022-12-15

**Authors:** E. Lin, Hans Garmo, Emil Hagström, Mieke Van Hemelrijck, Jan Adolfsson, Pär Stattin, Björn Zethelius, Danielle Crawley

**Affiliations:** 1 School of Cancer and Pharmaceutical Sciences, Translational Oncology and Urology Research (TOUR), King’s College London, London, UK; 2 Department of Surgical Sciences, Uppsala University, Uppsala, Sweden; 3 Department of Medical Sciences, Cardiology, Uppsala University, Uppsala, Sweden; 4 Uppsala Clinical Research Centre, Uppsala, Sweden; 5 Department of Clinical Science, Intervention and Technology, Karolinska Institute, Stockholm, Sweden; 6 Department of Public Health/Geriatrics, Uppsala University, Uppsala, Sweden

**Keywords:** Cancer epidemiology, Drug regulation, Hormonal therapies, Preventive medicine, Prostate cancer

## Abstract

**Background:**

Gonadotropin-releasing hormone agonists (GnRH) used in prostate cancer (PCa) are associated with atherogenic dyslipidaemia. It can be assumed that GnRH need to be used with greater caution in men with type 2 diabetes mellitus (T2DM). This study investigated association of GnRH with atherogenic lipids (AL) in PCa men with T2DM.

**Methods:**

Two cohorts including 38,311 men with 11 years follow-up based on Swedish national registers were defined (PCa-Exposure cohort and GnRH-Exposure cohort). Based on European guidelines on cardiovascular diseases (CVD), primary outcomes were defined as: 1.0 mmol/L increase in AL and lipid-lowering therapy (LLT) intensification. We used Cox proportional-hazards models and Kaplan–Meier curves to assess the association.

**Results:**

There was an association between GnRH and increased AL (i.e., triglyceride, PCa-Exposure cohort: HR 1.77, 95% CI 1.48–2.10; GnRH-Exposure cohort: HR 1.88, 95% CI 1.38–2.57). There was also an association between PCa diagnosis and increased AL. In contrast, no association between LLT intensification and GnRH was found.

**Conclusion:**

In this large population-based study, men with T2DM on GnRH for PCa had an increased risk of increased atherogenic lipids. These results highlight the need to closely monitor lipids and to be ready to intensify lipid-lowering therapy in men with T2DM on GnRH for PCa.

## Background

Prostate cancer (PCa) is the second most common cancer in men worldwide, with over a million new cases in 2019 [1]. Cardiovascular disease (CVD) is the leading cause of death in men with PCa. To prevent CVD, existing guidelines emphasise the importance of management of metabolic risk factors, including dyslipidaemia, especially in men with type 2 diabetes mellitus (T2DM) [2–4]. Dyslipidaemia is one of the most frequent comorbid conditions in cancer patients, about half of men with PCa also have increased atherogenic lipid levels, such as increased low-density lipoprotein cholesterol (LDL) and triglyceride (TG) levels [5–8]. Understanding how the management of dyslipidaemia and changes of atherogenic lipids over time is important to improve the CVD outcomes in men with PCa, especially in men with T2DM.

Moreover, elevated cholesterol may affect PCa progression through inflammation, steroidogenesis, and regulation of genes [7–9]. Nevertheless, although many studies have been investigated the impact of dyslipidaemia in PCa risk and progression, the results of these studies have been inconsistent [10–12], indicating that further studies are needed.

Use of Gonadotropin-releasing hormone agonists (GnRH) is associated with abnormal atherogenic lipid levels including increased low-density lipoprotein and triglyceride [3, 9–11]. Meanwhile, GnRH use is also associated with increased risk of T2DM, which is a major risk factor for CVD. Therefore, it is important to understand the long-term effects of GnRH used in PCa on atherogenic lipid levels, especially in men with pre-existing T2DM [12]. Nevertheless, there is little data on this association in men with T2DM. Such evidence is crucial to inform management of dyslipidaemia, with an overall goal of reducing risk of CVD in men with PCa treated with GnRH, especially in men with T2DM.

This study aimed to assess the association between GnRH use/PCa diagnosis and worsening dyslipidaemia through investigating the changes of atherogenic lipid levels, cholesterol ratio and the escalation of lipid-lowering therapy (LLT) in men with T2DM and PCa on/not on GnRH in the nationwide population-based cohorts.

## Methods

### Data source

We included men registered between 1 January 2006 and 31 December 2016 in the Swedish National Diabetes Register (NDR) who also had a PCa diagnosis based on the data in the Prostate Cancer data Base Sweden 4.1 (PCBaSe). The NDR was established in 1996, and currently enrols 90% of all T2DM cases in Sweden [13, 14]. The register contains longitudinal data on glycemic control (HbA1c), changes of antidiabetic treatment, and other clinical characteristics on diabetes [13]. Data on the use of LLT and lipid levels has also been collected in the NDR [15]. LLT is reported in over 95% of all patients in the NDR, and lipid levels are in about 75% of all patients [15].

Then, we also obtained data on PCa diagnosis, levels of prostate-specific antigen (PSA), stages and grades of PCa, Gleason score, dispensed medications, co-morbidities, and socioeconomic information from the PCBaSe, which is based on the National Prostate Cancer Register (NPCR) of Sweden [16, 17] that captures 98% of men diagnosed with PCa in Sweden [18]. In 2008, the NPCR was linked to other nationwide registers, including the National Patient Register, the Swedish Cancer Register, the Swedish Prescribed Drug Register, the Cause of Death Register, and the Longitudinal database on socioeconomic factors (LISA), by using the Swedish personal identification number, which provided prescribed drugs data and demographic information in our study [16, 17].

### Study population

To evaluate the association of GnRH and a PCa diagnosis per se with atherogenic lipids and LLT, we created two cohorts: “PCa-Exposure cohort” (men with PCa on or not on GnRH vs. PCa-free men) and “GnRH-Exposure cohort” (men with PCa receiving GnRH vs. men with PCa but not receiving GnRH) (Supplementary Fig. 1).

#### PCa-Exposure cohort

In the PCa-Exposure cohort, we selected men with T2DM who had at least four NDR registration data and were diagnosed with PCa on/not on GnRH after their third registered date in NDR (exposed men). The start of follow-up of this cohort was the date of PCa diagnosis. For each exposed man in this cohort, five men with T2DM but without PCa (non-exposed men) were randomly selected from NDR. The non-exposed men were matched to corresponding exposed men in this cohort on number of NDR registrations before inclusion and average time between NDR visits. Start of follow-up for each non-exposed man was inherited from the exposed man (Supplementary Fig. 1).

#### GnRH-Exposure cohort

The GnRH-Exposure cohort included men with T2DM who had at least four NDR registrations data and were diagnosed with PCa and treated with GnRH after the third NDR registration (exposed men). The start of follow-up was the date of the first filled prescription for GnRH. This cohort also included five men with T2DM and PCa but not on GnRH for each man treated with GnRH (non-exposed men). The non-exposed men were randomly selected from the NDR and matched to corresponding exposed men in this cohort based on the number of previous NDR registrations and average time between NDR visits. Start of follow-up for these non-exposed men was inherited from the corresponding exposed men in this cohort (Supplementary Fig. 1).

PCa-Exposure cohort enabled us to explore the association between PCa diagnosis per se and worsening dyslipidaemia. Additionally, we grouped exposed men in this cohort into (1) men with PCa on GnRH and (2) men with PCa but not on GnRH. It allowed us to examine the relationship between the use of GnRH and worsening dyslipidaemia as a first step in this cohort, through making comparison between men with PCa on GnRH and non-exposed men. Nevertheless, this estimated association might be affected by PCa diagnosis in this cohort. Meanwhile, the health seeking behaviours may be differed between men with PCa on GnRH and non-exposed men in the PCa-Exposure cohort, which might affect findings on the association between the use of GnRH and dyslipidaemia. Therefore, we further created the GnRH-Exposure cohort with the primary aim to investigate the association of lifelong use of GnRH and dyslipidaemia. The GnRH-Exposure cohorts precluded the potential impact of PCa diagnosis and reduced the impact of difference in healthcare seeking behaviours by matching non-exposed men to exposed men on the number of previous NDR registrations and average time between NDR visits.

In each cohort, we included men with at least one NDR registration in the follow-up, aiming to maximise use of data from the NDR. Meanwhile, to reduce the selection bias caused by the missing data at baseline, we include three NDR registrations prior to the start of follow-up as baseline characteristics and used last observation carried forward to impute the missing data at baseline (see below). Given above, in each cohort, we included men with at least four NDR registrations. The data on baseline characteristics was collected from the three last NDR records prior to the start of follow-up.

### Exposures

The primary exposure of the study was use of GnRH. When defining the use of GnRH as exposure in both cohorts, we excluded prescriptions that were part of a radical radiotherapy treatment by combining information on usage of GnRH from the Swedish Prescribed Drug Register and information on duration of neoadjuvant and adjuvant GnRH treatment in relation to radical radiotherapy recorded in the NPCR. Additionally, a study on a similar group of men showed good adherence to GnRH [19]. Hence, the use of GnRH referred to a lifelong treatment in this cohort.

Moreover, we also collected information on PCa diagnosis and risk categories of PCa from the NPCR, which were secondary exposures in our study as well. According to the National Comprehensive Cancer Network (NCCN), PCa risk categories are defined as following: Low-risk category: T1 or T2a stage, prostate-specific antigen (PSA) < 10 ng/mL, and Gleason score 6; Intermediate-risk category: T2b or T2c stage, 10 ng/mL, PSA < 20 ng/mL, or Gleason score 7; High-risk category: T3 or T4 stage, PSA ≥ 20 ng/mL, or Gleason score ≥8; Regional metastases category: any T, N1 and M0 stage; Distant metastases category: any T or N and M1 stage [20].

### Outcomes

The European Society of Cardiology and the European Atherosclerosis Society (ESC/EAS) guidelines state lipid level changes, such as the increase in LDL and the decreased in HDL, are related to cardiovascular events [21]. For instance, the ESC/EAS guidelines state that every 1.0 mmol/L reduction in LDL is associated with a corresponding 22% reduction in CVD mortality and morbidity [21, 22]. The guidelines also suggest to escalate the LLT if the current treatment is insufficient [21]. Therefore, based on the guideline and previous publications, following outcomes were chosen to represent worsening control of dyslipidaemia [21–25]:

1. There was an increase in level of LDL ≥ 1.0 mmol/L.
2. There was an increase in level of TG ≥ 1.0 mmol/L.
3. There was an increase in level of non-high-density lipoprotein cholesterol (non-HDL) ≥ 1.0 mmol/L.
4. HDL reduced to 1.1 mmol/L; or HDL was 10% lower than baseline measurement.
5. There was a more than 20% rise in the ratio non-HDL-to-HDL (non-HDL:HDL).
6. LLT initiation or intensified LLT. Intensified LLT was defined as a stepwise increase in statin intensity from low (simvastatin <20 mg, fluvastatin <20–40 mg, pravastatin <40 mg) to medium (simvastatin 20–<80 mg, fluvastatin 80 mg, atorvastatin 10–<40 mg, rosuvastatin 5–<20 mg) to high intensity (simvastatin ≥80 mg, atorvastatin ≥40 mg, rosuvastatin ≥20 mg) or addition of ezetimibe.

### Data analysis

For each outcome-related baseline variable (including LDL, TG, non-HDL, HDL, total cholesterol, non-HDL:HDL, and LLT), the last observation carried forward method was used to handle missing data at baseline. For example, for each man, if the last observation for each baseline variable (e.g., LDL) in the NDR was missing, the information was retrieved from the second last. If the second last observation was still missing, we used the data from the third last. If all the last three NDR observation were missing, we classified the outcome-related variable as missing (e.g., LDL) and excluded them with missing data when using Cox proportional hazard model to analyse the related outcome (e.g., LDL increased 1.0 mmol/L) (Supplementary Table 2).

Kaplan-Meier (KM) curves were firstly used to illustrate crude cumulative incidence of increase in atherogenic lipids and escalation of LLT. Additionally, we also used line graph to present measurement changes for different lipid levels (LDL, non-HDL, TG, HDL, non-HDL:HDL and total cholesterol) over time. The mean value of each lipid level for all men was calculated every three months from a half year prior to the start of follow-up to 2 years after the start of the follow up. The mean value of lipid level was determined through linear interpolation of the two adjacent lipid level values, which assumed a linear relationship between two consecutive values. The two adjacent lipid level values were on both sides of the time point of every three months from the start of follow-up.

Then, Cox proportional hazards regressions models were used to obtain hazards rations (HR) and 95% confidence interval (CI) for increase in atherogenic lipids (such as TG and LDL) and the escalation of LLT. Before applying Cox proportional hazards regression models, statistical tests and graphic diagnostics have been conducted, showing that no evidence supported the proportional hazards assumption was violated. All models were adjusted for age at PCa diagnosis, physical activity level, smoking, body mass index (BMI, kg/m^2^), systolic blood pressure (SBP), diastolic blood pressure (DBP), number of blood pressure medications, haemoglobin A1C (HbA1c), primary treatment of diabetes medication, duration of diabetes, educational level, civil status, Charlson Comorbidity Index (CCI), the number of NDR visits and the average time between two NDR visits.

All statistical analyses were performed using R 3.5.2 (R Foundation for Statistical Computing) and Statistical Analysis Systems release 9.4 (SAS Institute, Cary, NC).

The study was approved by the Research Ethics Board at Uppsala University, Sweden.

## Results

The PCa-Exposure cohort included 5714 men diagnosed with PCa on/not on GnRH (exposed men) and 28,445 men without PCa (non-exposed men), whereas the GnRH-Exposure cohort included 692 men with on GnRH (exposed men) and 3460 men with PCa but not on GnRH (non-exposed men). Both groups in each cohort had similar baseline characteristics, including age, education level, civil status, CCI, smoking habits, BMI, physical activity, T2DM status, blood pressure status, and lipid level status (Table 1 and Supplementary Table 1).

**Table 1:** Baseline characteristics of men in The National Diabetes Register in Sweden diagnosed with prostate cancer and/or used GnRH between 2006 and 2016 and their matched comparison.

	PCa-Exposure cohort				GnRH-Exposure cohort			
	Men with PCa *N* = 5714		PCa-free men *N* = 28,445		Men with PCa on GnRH *N* = 692		Men with PCa not on GnRH *N* = 3460	
	*N*	%	*N*	%	*N*	%	*N*	%
*Patients’ characteristics*								
Age (year)								
Median (IQR)	72.0 (67.0–78.0)		73.0 (68.0–79.0)		78.0 (72.0–83.0)		74.0 (69.8–79.3)	
CCI, *n* (%)								
0	2625	45.9	11,253	39.6	225	32.5	1062	30.7
1	1559	27.3	7853	27.6	210	30.3	1168	33.8
2	673	11.8	3739	13.1	94	13.6	498	14.4
3+	857	15.0	5600	19.7	163	23.6	732	21.2
Smoking, *n* (%)								
No	4581	80.2	22,273	78.3	524	75.7	2683	77.5
Yes	553	9.7	2912	10.2	51	7.4	267	7.7
Missing	580	10.2	3260	11.5	117	16.9	510	14.7
BMI, *n* (%)								
Median (IQR)	28.3 (25.7–31.2)		28.4 (25.8–31.5)		28.2 (25.8–31.1)		27.9 (25.5–30.9)	
*Health care seeking behaviour*								
The number of NDR visits (*n*)								
Median (IQR)	7.0 (4.0–11.0)		7.0 (4.0–11.0)		6.0 (4.0–10.0)		6.0 (4.0–10.0)	
The average time between NDR visits (days)								
Median (IQR)	237.8 (152.7–358.0)		245.3 (159.4–362.3)		186.1 (120.1–296.0)		186.9 (122.2–269.0)	
*T2DM status*								
Duration of T2DM, *n* (%)								
<10	2751	48.1	12,755	44.8	320	46.2	1703	49.2
10–<20	1939	33.9	10,149	35.7	222	32.1	1139	32.9
20–<30	530	9.3	3123	11.0	78	11.3	310	9.0
≥30	158	2.8	921	3.2	23	3.3	90	2.6
Missing	336	5.9	1497	5.3	49	7.1	218	6.3
HbA1c (mmol/mol),								
Median (IQR)	51.0 (45.0–59.0)		53.0 (46.0–62.0)		51.0 (45.0–58.5)		51.0 (45.0–60.0)	
Treatment of T2DM, *n* (%)								
Diet controlled	1333	23.3	6196	21.8	195	28.2	867	25.1
Oral hypoglycaemics	2513	44.0	12,003	42.2	260	37.6	1513	43.7
Insulin	1868	32.7	10,246	36.0	237	34.2	1080	31.2
*PCa status*								
PCa diagnosis								
No PCa	–	–	28,445	100	–	–	–	–
PCa	5714	100	–	–	692	100	3460	100
Using GnRH, *n* (%)								
No PCa	–	–	28,445	100	–	–	–	–
PCa without GnRH	4274	74.8	–	–	–	–	3460	100
PCa with GnRH	1400	25.2	–	–	692	100	–	–
PCa risk group								
No PCa	–	–	28,445	100	–	–	–	–
Low risk	1122	19.8	–	–	145	21.0	1437	41.5
Intermediate risk	1838	32.2	–	–	229	33.1	1272	36.8
High risk	1531	26.8	–	–	232	33.5	533	15.4
Regional metastases	389	6.8	–	–	42	6.1	56	1.6
Distance metastases	650	11.4	–	–	32	4.6	39	1.1
Missing data	184	3.2	–	–	12	1.7	123	3.6
*Blood pressure status*								
SBP (mmHg)								
Median ± IQR	135.0 (125.0–145.0)		135.0 (125.0–145.0)		134.0 (125.0–142.0)		135.0 (125.0–145.0)	
DBP (mmHg)								
Median ± IQR	77.0 (70.0–80.0)		75.0 (70.0–80.0)		72.0 (68.0–80.0)		75.0 (70.0–80.0)	
Numbers of blood pressure drugs, No. (%)								
0	867	15.2	4212	14.8	110	15.9	496	14.3
1	1353	23.7	6674	23.5	164	23.7	911	26.3
2	1702	29.8	8308	29.2	198	28.6	1007	29.1
3	1300	22.8	6727	23.6	176	25.4	794	22.9
4+	492	8.6	2524	8.8	44	6.3	252	7.3
*Lipid levels*								
Non-HDL (mmol/L)								
Median ± IQR	3.1 (2.5–3.8)		3.1 (2.5–3.8)		3.0 (2.5–3.7)		3.1 (2.5–3.8)	
Total cholesterol (mmol/L)								
Median ± IQR	4.3 (3.7–5.0)		4.3 (3.7–5.0)		4.4 (3.8–5.0)		4.4 (3.8–5.1)	
Triglyceride (mmol/L)								
Median ± IQR	1.4 (1.0–2.0)		1.5 (1.1–2.1)		1.3 (1.0–1.8)		1.4 (1.0–2.0)	
LDL (mmol/L)								
Median ± IQR	2.4 (1.9–3.0)		2.3 (1.8–3.0)		2.3 (1.9–3.0)		2.4 (1.9–3.0)	
HDL (mmol/L)								
Median ± IQR	1.1 (1.0–1.4)		1.1 (1.8–3.0)		1.2 (1.0–1.5)		1.2 (1.0–1.4)	
Non-HDL:HDL								
Median ± IQR	2.7 (2.0–3.6)		2.8 (2.1–3.7)		2.6 (2.2–3.7)		2.7 (2.2–3.8)	
Intensity of lipid-lowering therapy, *n* (%)^a^								
No drug	1992	34.9	9794	34.4	240	34.7	1193	34.5
Low	363	6.4	1773	6.2	42	6.1	187	5.4
Intermediate	2891	50.6	14,489	50.9	356	51.4	1759	50.8
High	359	6.3	1877	6.6	41	5.9	246	7.1
Ezetimibe	109	1.9	512	1.8	13	1.9	75	2.2

*PCa* prostate cancer, *GnRH* gonadotropin-releasing hormone agonists, *LDL* low-density lipoprotein cholesterol, *Non-HDL* non-high-density lipoprotein cholesterol, *TG* triglyceride, *NDR* National Diabetes Register, *BMI* body mass index, *BP* blood pressure, *SBP* systolic blood pressure, *DBP* diastolic blood pressure, *T2DM* type 2 diabetes mellitus, *HbA1c* haemoglobin A1C, *CCI* Charlson Comorbidity Index.

^a^Different lipid-lowering therapy’s level were defined as followings: No drug group: men without lipid-lowering therapy; Low-level group: used Simvastatin <20 mg, Fluvastatin <20–40 mg, or Pravastatin <40 mg; Intermediate level group: used Simvastatin 20–<80 mg, Fluvastatin 80 mg, Atorvastatin 10–<40 mg, or Rosuvastatin 5–<20 mg; High-level group: used Simvastatin ≥80 mg, Atorvastatin ≥40 mg, or Rosuvastatin ≥20 mg. Ezetimibe group in the table represented the number of patients with Ezetimibe with/without statins.

### PCa-Exposure cohort

**Fig. 1:**
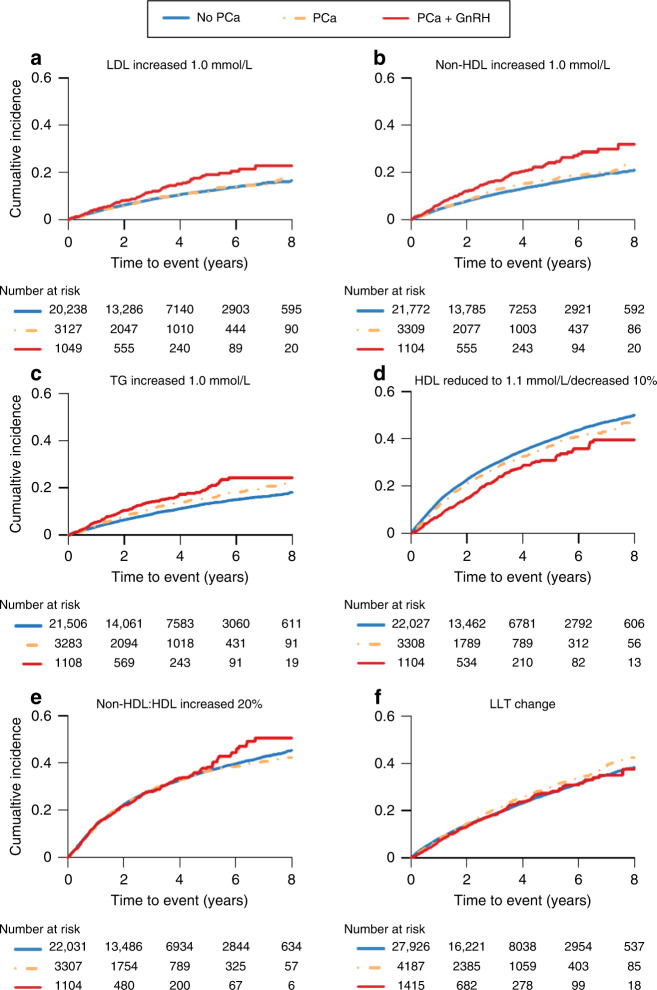
Kaplan–Meier curves of cumulative incidence of worsening control of lipid levels and escalation of lipid-lowering therapy by PCa status in PCa-Exposure cohort^1^. The figure showed a higher cumulative incidence for increase in LDL, non-HDL and TG with GnRH treatment, compared to men without PCa (**a**–**c**). Besides, we also observed that men with GnRH had a lower cumulative incidence of worsening control of HDL than PCa-free men (**d**–**f**), no changes of cumulative incidence for increase in non-HDL:HDL and the escalation of LLT were observed in PCa men with use of GnRH.^1^ In **a**, we excluded those men without data on LDL in NDR register. We excluded those men without data on total cholesterol and HDL in NDR register in **b**, **e**. In **c**, we excluded those men without data on TG in NDR register. We excluded those men without data on HDL in NDR register in **d**. In **f**, we excluded those men without data on use of stains or men with Ezetimibe. PCa prostate cancer, GnRH gonadotropin-releasing hormone agonists, LDL low-density lipoprotein cholesterol, non-HDL non-high-density lipoprotein cholesterol, TG triglyceride, LLT lipid-lowering therapy.

Figure 1 showed a higher cumulative incidence for increase in LDL, TG and non-HDL with GnRH treatment, compared to men without PCa (non-exposed men in this cohort). In Fig. 1, we also observed that men with PCa and on GnRH had a lower cumulative incidence of worsening control of HDL than PCa-free men (Fig. 1d). No changes of cumulative incidence for the increase in non-HDL:HDL and the escalation of LLT and were observed in men with PCa and on GnRH (Fig. 1e, f).

In Supplementary Fig. 2, changes of lipid levels, especially LDL, non-HDL and TG levels, in PCa men with GnRH were more obvious during the first 6 months of follow-up, compared with PCa-free men. The lipid levels in these men were higher than for PCa-free men during the period of follow-up after the first 6 months.

In Table 2, findings from adjusted Cox proportional hazard regression model supported the curded results from the KM curves. Table 2 showed the adjusted association between PCa diagnosis and increase in TG and non-HDL-C levels, compared men without PCa (TG: HR: 1.30, 95% CI: 1.08–1.47; non-HDL: HR: 1.22, 95% CI: 1.11–1.33). However, PCa diagnosis was associated with better control of HDL (HR: 0.86, 95% CI: 0.80–0.91). No association was found between PCa diagnosis and increased risk of worsening control of non-HDL:HDL (HR: 0.99, 95% CI: 0.93–1.05). No association between diagnosis of PCa and the LLT use (HR: 1.05, 95% CI: 0.98–1.12) was observed. When PCa men were grouped into different risk categories, the increased risk of worsening control of lipid levels was only found in men with regional or distant metastatic disease (Table 2). No association between escalation of LLT and PCa diagnosis were found across different PCa risk categories.

**Table 2:** HR and 95% CI for worsening control of lipid levels and escalation of lipid-lowering therapy by using different definitions of events in PCa-Exposure cohort.

	LDL increased 1.0 mmol/L^a^		Non-HDL increased 1.0 mmol/L^b^		TG increased 1.0 mmol/L^c^		HDL reduced to 1.1 mmol/L or decreased 10%^d^		Non-HDL: HDL increased 20%^e^		Lipid-lowering therapy change^f^	
*Crude model*												
PCa diagnosis												
No	1.00	ref.	1.00	ref.	1.00	ref.	1.00	ref.	1.00	ref.	1.00	Ref.
Yes	1.10	(0.99–1.23)	1.20	(1.10–1.32)	1.31	(1.19–1.44)	0.86	(0.80–0.91)	1.02	(0.95–1.09)	1.07	(1.00–1.14)
Using GnRH												
No PCa	1.00	ref.	1.00	ref.	1.00	ref.	1.00	ref.	1.00	ref.	1.00	ref.
PCa without GnRH	1.01	(0.89–1.14)	1.10	(0.99–1.22)	1.24	(1.11–1.38)	0.90	(0.84–0.97)	1.01	(0.93–1.09)	1.09	(1.02–1.18)
PCa with GnRH	1.42	(1.17–1.72)	1.58	(1.35–1.85)	1.57	(1.32–1.87)	0.70	(0.61–0.80)	1.06	(0.92–1.21)	0.97	(0.85–1.10)
PCa risk group												
No PCa	1.00	ref.	1.00	ref.	1.00	ref.	1.00	ref.	1.00	ref.	1.00	ref.
Low risk	0.95	(0.76–1.19)	1.18	(0.99–1.41)	1.37	(1.14–1.64)	0.93	(0.82–1.05)	1.03	(0.90–1.19)	1.20	(1.06–1.36)
Intermediate risk	1.08	(0.91–1.29)	1.13	(0.97–1.31)	1.23	(1.05–1.45)	0.96	(0.87–1.06)	1.02	(0.91–1.14)	1.13	(1.02–1.25)
High risk	1.07	(0.88–1.31)	1.11	(0.94–1.32)	1.15	(0.95–1.38)	0.74	(0.65–0.84)	0.97	(0.85–1.11)	0.99	(0.88–1.12)
Regional metastases	1.45	(1.00–2.09)	1.55	(1.14–2.11)	1.34	(0.93–1.93)	0.66	(0.51–0.87)	1.01	(0.77–1.33)	0.89	(0.69–1.16)
Distant metastases	1.52	(1.12–2.06)	1.66	(1.28–2.14)	1.77	(1.35–2.33)	0.69	(0.55–0.86)	1.00	(0.79–1.27)	0.80	(0.63–1.01)
Missing data	1.00	(0.57–1.77)	1.22	(0.78–1.91)	1.92	(1.28–2.87)	1.03	(0.76–1.40)	1.29	(0.93–1.79)	1.15	(0.84–1.57)
*Adjusted model*^g^												
PCa diagnosis												
No	1.00	ref.	1.00	ref.	1.00	ref.	1.00	ref.	1.00	ref.	1.00	Ref.
Yes	1.11	(0.99–1.23)	1.22	(1.11–1.33)	1.30	(1.08–1.47)	0.86	(0.80–0.91)	0.99	(0.93–1.05)	1.05	(0.98–1.12)
Using GnRH												
No PCa	1.00	ref.	1.00	ref.	1.00	ref.	1.00	ref.	1.00	ref.	1.00	ref.
PCa without GnRH	1.01	(0.89–1.14)	1.10	(0.99–1.22)	1.22	(1.09–1.36)	0.90	(0.84–0.97)	1.00	(0.93–1.08)	1.06	(0.98–1.13)
PCa with GnRH	1.45	(1.20–1.76)	1.64	(1.40–1.92)	1.77	(1.48–2.10)	0.71	(0.62–0.81)	1.07	(0.93–1.23)	1.02	(0.90–1.17)
PCa risk group												
No PCa	1.00	ref.	1.00	ref.	1.00	ref.	1.00	ref.	1.00	ref.	1.00	ref.
Low risk	0.94	(0.75–1.17)	1.18	(0.98–1.41)	1.30	(1.08–1.56)	0.92	(0.82–1.04)	1.02	(0.89–1.18)	1.11	(0.98–1.25)
Intermediate risk	1.09	(0.91–1.29)	1.13	(0.98–1.32)	1.21	(1.03–1.42)	0.95	(0.82–1.05)	1.01	(0.90–1.13)	1.08	(0.97–1.20)
High risk	1.09	(0.90–1.33)	1.14	(0.96–1.35)	1.24	(1.03–1.49)	0.75	(0.66–0.85)	0.99	(0.86–1.13)	1.03	(0.92–1.17)
Regional metastases	1.48	(1.03–2.14)	1.62	(1.19–2.20)	1.52	(1.05–2.19)	0.68	(0.52–0.89)	1.04	(0.79–1.36)	0.92	(0.71–1.20)
Distant metastases	1.54	(1.13–2.09)	1.71	(1.32–2.21)	2.03	(1.54–2.67)	0.70	(0.56–0.88)	1.01	(0.80–1.28)	0.85	(0.68–1.07)
Missing data	1.00	(0.57–1.77)	1.20	(0.76–1.89)	1.77	(1.18–2.65)	1.10	(0.75–1.39)	1.27	(0.91–1.76)	1.09	(0.80–1.50)

*HR* hazard ratio, *95% CI* 95% confidence interval, *PCa* prostate cancer, *GnRH* gonadotropin-releasing hormone agonists, *LDL* low-density lipoprotein cholesterol, *HDL* high-density lipoprotein cholesterol, *Non-HDL* non-high-density lipoprotein cholesterol, *TG* triglyceride, *NDR* National Diabetes Register.

^a^We excluded those men without data on LDL in NDR register.

^b^We excluded those men without data on total cholesterol and HDL in NDR register.

^c^We excluded those men without data on TG in NDR register.

^d^We excluded those men without data on HDL in NDR register.

^e^We excluded those men without data on total cholesterol and HDL.

^f^We excluded those men without data on use of stains or men with Ezetimibe.

^g^Adjusted for age, BMI, lifestyle, BP status, T2DM status, socioeconomic characteristics, CCI and health seeking behaviours, including age at PCa diagnosis, physical activity, smoking, BMI, SBP, DBP, blood pressure medication, HbA1c, diabetes medication, duration of diabetes, educational level, civil status, CCI, the number of NDR visits and the average time between two NDR visits.

When we grouped men with PCa into men with PCa on GnRH and men with PCa but not on GnRH, we found that men treated with GnRH had a higher risk of worsening control of LDL, TG and non-HDL levels, which belong to atherogenic lipids, compared to men without PCa (Table 2). No association was observed between use of GnRH and rise in non-HDL:HDL and escalation of LLT (Table 2).

### GnRH-Exposure cohort

**Fig. 2:**
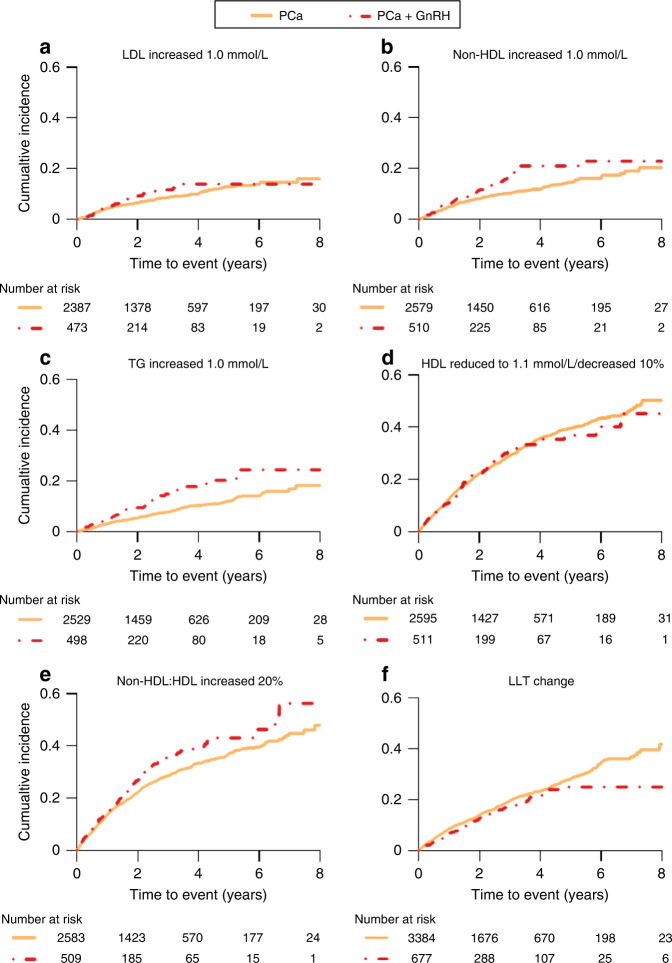
Kaplan–Meier curves for cumulative incidence of worsening control of lipid levels and escalation of lipid- lowering therapy by PCa status in GnRH-Exposure cohort^1^. In this figure, a higher cumulative incidence for worsening control of LDL, non-HDL, TG and non-HDL:HDL was observed in PCa men on GnRH, compared to PCa men without GnRH (**a**–**c**, **e**). However, there was no obvious change of cumulative incidence for the decrease in HDL and the escalation of LLT (**d**, **f**).^1^ In **a**, we excluded those men without data on LDL in NDR register. We excluded those men without data on total cholesterol and HDL in NDR register in **b**, **e**. In **c**, we excluded those men without data on TG in NDR register. We excluded those men without data on HDL in NDR register in **d**. In **f**, we excluded those men without data on use of stains or men with Ezetimibe. PCa prostate cancer, GnRH gonadotropin-releasing hormone agonists, LDL low-density lipoprotein cholesterol, non-HDL non-high-density lipoprotein cholesterol, TG triglyceride, LLT lipid-lowering therapy.

In line with results in the PCa-Exposure cohort, in Fig. 2, a higher cumulative incidence for worsening control of lipid levels was observed in PCa men on GnRH, compared to PCa men without GnRH (non-exposed men in GnRH-Exposure cohort). Men with GnRH had a lower cumulative incidence for worsening control of HDL (Fig. 2). There was no change of cumulative incidence for escalation of LLT (Fig. 2). In the first 6 months of follow-up, there was a rapid increase of lipid levels in PCa men with GnRH, particularly TG level, compared to PCa men without GnRH (Supplementary Fig. 3). The lipid levels in these men were always higher than for PCa men without GnRH during the first 6 months (Supplementary Fig. 3).

Table 3 included results from adjusted Cox proportional regression model in this cohort, showing the association between use of GnRH and worsening control of TG, non-HDL and non-HDL:HDL in PCa men on GnRH, compared to men with PCa but not on GnRH (Table 3). However, no association was seen between use of GnRH and worsening control of HDL and escalation of LLT (Table 3). Increased risk of worsening control of LDL level and better control of HDL were seen in men with metastatic disease (Table 3). In Table 3, a similar result was seen for other lipids, including non-HDL and TG. No differences by PCa risk categories were seen for the risk of worsening control of non-HDL:HDL and escalation of LLT.

**Table 3:** HR and 95% CI for worsening control of lipid levels and escalation of lipid-lowering therapy by using different definitions of events in GnRH-Exposure cohort.

	LDL increased 1.0 mmol/L^a^		Non-HDL increased 1.0 mmol/L^b^		TG increased 1.0 mmol/L^c^		HDL reduced to 1.1 mmol/L or decreased 10%^d^		Non-HDL:HDL increased 20%^e^		Lipid-lowering therapy change^f^	
*Crude model*												
Using GnRH												
PCa without GnRH	1.00	ref.	1.00	ref.		ref.	1.00	ref.	1.00	ref.	1.00	ref.
PCa with GnRH	1.23	(0.87–1.73)	1.49	(1.13–1.97)	1.79	(1.32–2.42)	0.98	(0.81–1.20)	1.22	(1.01–1.47)	0.81	(0.64–1.02)
PCa risk group												
Low risk	1.00	ref.	1.00	ref.	1.00	ref.	1.00	ref.	1.00	ref.	1.00	ref.
Intermediate risk	0.98	(0.73–1.32)	1.04	(0.80–1.34)	1.05	(0.79–1.40)	0.99	(0.85–1.15)	1.08	(0.93–1.26)	0.92	(0.78–1.10)
High risk	0.91	(0.63–1.32)	0.89	(0.65–1.24)	1.05	(0.74–1.48)	0.86	(0.71–1.05)	0.97	(0.80–1.18)	0.86	(0.69–1.07)
Regional metastases	0.52	(0.16–1.64)	0.66	(0.27–1.60)	0.68	(0.25–1.85)	0.55	(0.31–0.98)	0.80	(0.48–1.34)	0.50	(0.26–0.97)
Distance metastases	1.64	(0.60–4.47)	1.67	(0.73–3.78)	0.80	(0.20–3.25)	1.43	(0.80–2.54)	1.45	(0.82–2.58)	1.19	(0.67–2.12)
Missing data	1.48	(0.77–2.84)	1.42	(0.80–2.52)	0.68	(0.28–1.67)	1.04	(0.70–1.54)	0.90	(0.60–1.37)	0.78	(0.48–1.28)
*Adjusted model*^g^												
Using GnRH												
PCa without GnRH	1.00	ref.	1.00	ref.	1.00	ref.	1.00	ref.	1.00	ref.	1.00	ref.
PCa with GnRH	1.18	(0.83–1.67)	1.49	(1.12–1.99)	1.88	(1.38–2.57)	0.98	(0.80–1.20)	1.20	(1.00–1.45)	0.88	(0.70–1.11)
PCa risk group												
Low risk	1.00	ref.	1.00	ref.	1.00	ref.	1.00	ref.	1.00	ref.	1.00	ref.
Intermediate risk	0.99	(0.74–1.34)	1.03	(0.80–1.34)	1.10	(0.82–1.47)	0.98	(0.84–1.14)	1.07	(0.92–1.25)	0.95	(0.80–1.13)
High risk	0.85	(0.58–1.25)	0.87	(0.62–1.22)	1.14	(0.79–1.63)	0.83	(0.68–1.02)	0.98	(0.80–1.20)	0.94	(0.76–1.18)
Regional metastases	0.49	(0.15–1.55)	0.70	(0.28–1.72)	0.75	(0.27–2.07)	0.54	(0.30–0.96)	0.83	(0.49–1.40)	0.53	(0.27–1.03)
Distant metastases	1.68	(0.61–4.66)	1.66	(0.72–3.84)	0.86	(0.21–3.54)	1.48	(0.83–2.66)	1.57	(0.88–2.82)	1.33	(0.74–2.39)
Missing data	1.55	(0.79–3.03)	1.54	(0.86–2.77)	0.89	(0.36–2.20)	1.06	(0.71–1.57)	0.99	(0.65–1.50)	0.86	(0.52–1.41)

*HR* hazard ratio, *95% CI* 95% confidence interval, *PCa* prostate cancer, *GnRH* gonadotropin-releasing hormone agonists, *LDL* low-density lipoprotein cholesterol, *HDL* high-density lipoprotein cholesterol, *Non-HDL* non-high-density lipoprotein cholesterol, *TG* triglyceride, *NDR* National Diabetes Register.

^a^We excluded those men without data on LDL in NDR register.

^b^We excluded those men without data on total cholesterol and HDL in NDR register.

^c^We excluded those men without data on TG in NDR register.

^d^We excluded those men without data on HDL in NDR register.

^e^We excluded those men without data on total cholesterol and HDL.

^f^We excluded those men without data on use of stains or men with Ezetimibe.

^g^Adjusted for age, BMI, lifestyle, BP status, T2DM status, socioeconomic characteristics, CCI and health seeking behaviours, including age at PCa diagnosis, physical activity, smoking, BMI, SBP, DBP, blood pressure medication, HbA1c, diabetes medication, duration of diabetes, educational level, civil status, CCI, the number of NDR visits and the average time between two NDR visits.

## Discussion

In this nationwide, population-based study, men with T2DM on GnRH for PCa had a worsening control of atherogenic lipid levels. However, there was no association between use of GnRH and escalation of LLT, which suggested that treatment for lowering atherogenic lipid levels in men with T2DM on GnRH for PCa might not rigorous.

### GnRH and atherogenic lipids

We demonstrated an association between GnRH and lipid control without the effect of PCa diagnosis per se in men with T2DM, which is in line with previous epidemiological and clinical studies [3, 26–28]. These previous studies revealed a higher risk of development of worsening dyserlipidaemia, in PCa men receiving GnRH [3, 26–28]. Our study also indicated that 6 months of GnRH was significantly associated with increasing lipid levels, especially atherogenic lipids (i.e., LDL and TG). Thereafter levels remained higher compared to with T2DM but not on GnRH. These findings are supported by existing smaller prospective studies but only with short follow-up periods of 6–12 months showing increases in total cholesterol, LDL and TG in men on GnRH [10, 29–31]. Nevertheless, in contrast to worsening dyslipidaemia, HDL level was higher in PCa men with GnRH over time in our study, compared to men without PCa, indicating that use of GnRH was associated with better control of HDL. This finding agreed with previous observational studies with a significant increase in HDL in patients on GnRH agonists [29, 30, 32]. For cholesterol ratio, non-HDL:HDL has been shown to be a risk factor for cardiovascular diseases [25]. Our study showed an association between use of GnRH and increased non-HDL:HDL. This finding was supported by the review conducted by Zareba et al. in 2016 [33].

For the underlying mechanisms of this association between the use of GnRH and increased atherogenic lipid levels, given that GnRH lower testosterone levels by continually stimulating the GnRH receptor, it is suggested that low levels of testosterone are associated with decreased TG turnover and alterations in lipoprotein lipase enzyme activity [34]. Subsequently, abnormal levels of LDL and TG occur in men with GnRH [34]. Meanwhile, a study conducted by Smith et al. found that short-term treatment with GnRH significantly decreased insulin sensitivity in PCa men [31]. Decreased insulin sensitivity has been observed to increase atherogenic lipid levels, such as TG and LDL levels [27, 35, 36]. However, underlying mechanisms to explain the improvement in HDL are not clear and warrant further study.

Besides, no association was found between use of GnRH and the escalation of LLT in our study. It may be explained by, if worsening control of atherogenic lipid levels is present in PCa men on GnRH, that diet and lifestyle changes are recommended as first-line interventions to improve lipids level [27], instead of use or escalation in LLT.

### PCa diagnosis and atherogenic lipids

PCa and dyserlipidaemia are common conditions, and often occur in the same man. Numerous studies have investigated the association between levels of cholesterol and PCa development and progression. Although some studies reported no association between PCa risk and lipid profiles (including total cholesterol, TG, HDL, LDL and the cholesterol ratio) [37–41], many studies showed that elevated lipid levels (especially atherogenic lipid levels) were associated with increased risk of PCa diagnosis and the progression and development of advanced PCa [37, 42–44]. Various animal and in vitro experiments proposed that elevated lipids level trigger PCa development and progression by inflammation and regulation of genes [43, 45, 46]. The increased level of insulin plays a crucial role on the association between PCa and elevated lipid levels, which has ubiquitous effects in vivo and triggers cascades of numerous signal transduction pathways, such as P13K/AKT, mTOR, and COX-2 [43, 45, 47, 48]. Nevertheless, the exact underlying mechanism of the association remains poorly understood.

Notably, little evidence is available into the impact of PCa diagnosis per se on the dyslipidaemia. Our study found an association of PCa diagnosis with worsening control of TG and non-HDL levels, compared to PCa-free men, in the PCa-Exposure cohort. Nevertheless, the increased risk of worsening control of non-HDL level was only found in men with regional or distant metastatic disease in PCa-Exposure cohort. The association between PCa diagnosis and worsening control of TG levels was shown across all PCa risk categories. Besides, we also found better control of HDL in PCa men in the PCa-Exposure cohort. These findings of the increase in lipids levels in PCa men may be explained by that lipogenesis is a fundamental aspect of PCa cell biology [49–51]. They found the overexpression of lipogenic proteins and enzymes in the PCa cells resulting in lipid accumulation [49–51], which might be affected by the androgens and dysregulated androgen receptor function [46]. Nevertheless, the exactly underlying mechanism of the association between PCa diagnosis and dyslipidaemia warrants furthers study.

## Strengths and limitations

To our knowledge, this is the largest population-based cohort study examining this association with up to 11 years of follow-up. Secondly, it included detailed longitudinal data within the NDR and PDR, therefore, we were able to look at the different kinds of lipids, including atherogenic lipid levels and the cholesterol ratio, and escalation of LLT, and observed their changes over time. Thirdly, by matching cases and relevant comparisons on the number of NDR visits and average time between two NDR visits, we reduced the impact of NDR visits behaviours, which is likely to be correlated with patient compliance and the quality of diseases management.

One limitation of the study is that approximately 3–6% of men had missing data at baseline measurement. These missing data were imputed by using last observation carried forward, which might underestimate the effect of exposures. Additionally, to maximise use of the available data and reduce selection bias, we used the last observation carried forward to impute the missing data at baseline. Nevertheless, it is not possible to completely exclude selection bias as we excluded men still with missing data in both cohorts (Supplementary Table 2), although the proportions were small after using last observation carried forward imputation method. Furthermore, despite many confounders that have been adjusted in the statistic model, due to lack of information, several residual confounding, like family history of dyslipidaemia and PCa, cannot be excluded. Both cohorts also included a very limited number of men receiving other androgen-deprivation therapies (ADTs) including orchiectomy and androgen receptor targeted drugs. As these numbers were very small, the impact of other ADTs on the association between GnRH and atherogenic lipids was ignored in the current study. Nevertheless, the potential effects of other ADTs on this association as well as the impact of other ADTs on atherogenic lipids warrants further study.

## Conclusions

This large population-based cohort study showed that treatment of GnRH was associated with increased levels in atherogenic lipids, such as LDL and TG, but not with the escalation in lipid-lowering therapy. These findings support the previous data indicating the association between GnRH and increased risk of CVD, and highlight the need to closely monitor lipid levels and to be ready to intensify lipid-lowering therapy in men with T2DM and PCa starting treatment with GnRH.

## Supplementary information

Supplementary table 1. Additional patients’ characteristics of men in NDR diagnosed with prostate cancer and/or used GnRH between 2006 and¬ 2016 and their matched comparison

Supplementary table 2. Number of events and the number of men excluded for having missing data on each outcome-related variable in PCa-Exposure cohort and GnRH-Exposure cohort

Supplementary figure 1. Patient inclusion and exclusion flowchart

Supplementary figure 2. Lipid levels change over time in PCa-Exposure cohort

Supplementary figure 3. Lipid levels change over time in GnRH-Exposure cohort

aj-checklist_ BJC-A3339738R

## Data Availability

The data that support the findings of this study are available from PCBaSe Sweden, but restrictions apply to the availability of these data, which were used under license for the current study, and so are not publicly available. Data are, however, available from the authors upon reasonable request and with permission of PCBaSe Sweden.
