# Supplementary material for: Association between atherogenic lipids and GnRH agonists for prostate cancer in men with T2DM: a nationwide, population-based cohort study in Sweden
Source: Br J Cancer. 2022 Dec 15;128(5):814–24. doi: 10.1038/s41416-022-02091-z (PMC9977763; doi:10.1038/s41416-022-02091-z)
Supplement: Supplementary file 1 — Supplementary table 1. Additional patients’ characteristics of men in NDR diagnosed with prostate cancer and/or used GnRH between 2006 and¬ 2016 and their matched comparison [file 41416_2022_2091_MOESM1_ESM.docx]

**Supplementary table 1. Additional patients’ characteristics of men in NDR diagnosed with prostate cancer and/or used GnRH between 2006 and­ 2016 and their matched comparison**

|  | **PCa-Exposure cohort** | | |  | **GnRH-Exposure cohort** | | | | |
| --- | --- | --- | --- | --- | --- | --- | --- | --- | --- |
|  | **men with PCa**  N=5,714 | | **PCa-free men**  N=28,445 | | **men with PCa on GnRH**  N=692 | | **men with PCa but not on GnRH**  N=3,460 | |  |
|  | N | % | N | % | N | % | N | % |  |
| **Education level, n (%)** |  |  |  |  |  |  |  |  |  |
| Low | 2,340 | 41.0 | 12,231 | 43.0 | 308 | 44.5 | 1,333 | 38.5 |  |
| Middle | 2,755 | 48.2 | 13,172 | 46.3 | 297 | 42.9 | 1,678 | 48.5 |  |
| High | 570 | 10.0 | 2,686 | 9.4 | 82 | 11.8 | 432 | 12.5 |  |
| Missing | 49 | 0.9 | 356 | 1.3 | 5 | 0.7 | 17 | 0.5 |  |
| **Civil status, n (%)** |  |  |  |  |  |  |  |  |  |
| Married | 3,708 | 64.9 | 17,582 | 61.8 | 444 | 64.2 | 2,248 | 65.0 |  |
| Not married (+Divorced/Widower/missing) | 2,006 | 35.1 | 10,863 | 38.2 | 248 | 35.8 | 1,212 | 35.0 |  |
| **Physical activity (%) ^1^** |  |  |  |  |  |  |  |  |  |
| Daily | 573 | 10.0 | 3,452 | 12.1 | 98 | 14.2 | 380 | 11.0 |  |
| 3-5 times a week | 500 | 8.8 | 2,486 | 8.7 | 69 | 10.0 | 268 | 7.7 |  |
| 1-2 times a week | 902 | 15.8 | 4,261 | 15.0 | 96 | 13.9 | 503 | 14.5 |  |
| Less than once a week | 1,016 | 17.8 | 4,786 | 16.8 | 104 | 15.0 | 629 | 18.2 |  |
| Never | 1,531 | 26.8 | 6,919 | 24.3 | 140 | 20.2 | 784 | 22.7 |  |
| Missing | 1,192 | 20.9 | 6,541 | 23.0 | 185 | 26.7 | 896 | 25.9 |  |
| **Number of clinic visits, n (%)** |  |  |  |  |  |  |  |  |  |
| 3 - <10 | 3,794 | 66.4 | 18,879 | 66.4 | 504 | 72.8 | 2,520 | 72.8 |  |
| 10 - <20 | 1,520 | 26.6 | 7,578 | 26.6 | 151 | 21.8 | 755 | 21.8 |  |
| 20 - <30 | 315 | 5.5 | 1,567 | 5.5 | 29 | 4.2 | 145 | 4.2 |  |
| $\geq$30 | 85 | 1.5 | 421 | 1.5 | 8 | 1.2 | 40 | 1.2 |  |

^1^ In the NDR, once physical activity is defined as at least 30 minutes of walking or similar activity.

*Abbreviations: PCa=prostate cancer; GnRH= Gonadotropin-releasing hormone agonists; NDR= national diabetes register.*
