# Supplementary material for: Association between atherogenic lipids and GnRH agonists for prostate cancer in men with T2DM: a nationwide, population-based cohort study in Sweden
Source: Br J Cancer. 2022 Dec 15;128(5):814–24. doi: 10.1038/s41416-022-02091-z (PMC9977763; doi:10.1038/s41416-022-02091-z)
Supplement: Supplementary file 2 — Supplementary table 2. Number of events and the number of men excluded for having missing data on each outcome-related variable in PCa-Exposure cohort and GnRH-Exposure cohort [file 41416_2022_2091_MOESM2_ESM.docx]

**Supplementary table 2 Number of events and the number of men excluded for having missing data on each outcome-related variable in PCa-Exposure cohort and GnRH-Exposure cohort**

|  | **PCa-Exposure cohort** | | | | **GnRH-Exposure cohort** | | | |
| --- | --- | --- | --- | --- | --- | --- | --- | --- |
|  | **men with PCa**  N=5,714 | | **PCa-free men**  N=28,445 | | **men with PCa on GnRH**  N=692 | | **men with PCa but not on GnRH**  N=3,460 | |
|  | N | % | N | % | N | % | N | % |
| **LDL increased 1.0 mmol/L ^1^** |  |  |  |  |  |  |  |  |
| No | 3,782 | 90.5 | 18,387 | 90.8 | 434 | 91.6 | 2,192 | 91.8 |
| Yes | 397 | 9.5 | 1,853 | 9.2 | 40 | 8.4 | 195 | 8.2 |
| Total number of patients in the subset | 4,179 | 100.0 | 20,240 | 100.0 | 474 | 100.0 | 2,387 | 100.0 |
| Number of men excluded for missing data on LDL | 1,535 | - | 8,205 | - | 218 | - | 1,073 | - |
| **TG increased 1.0 mmol/L ^2^** |  |  |  |  |  |  |  |  |
| No | 3,882 | 88.3 | 19,446 | 90.4 | 391 | 78.4 | 2,071 | 81.9 |
| Yes | 513 | 11.7 | 2,064 | 9.6 | 108 | 21.6 | 458 | 18.1 |
| Total number of patients in the subset | 4,395 | 100.0 | 21,510 | 100.0 | 499 | 100.0 | 2,529 | 100 |
| Number of men excluded for missing data on TG | 1,319 | - | 6,935 | - | 193 | - | 931 | - |
| **Non-HDL increased 1.0 mmol/L ^3^** |  |  |  |  |  |  |  |  |
| No | 3,847 | 87.1 | 19,303 | 88.6 | 449 | 87.9 | 2,328 | 90.3 |
| Yes | 570 | 12.9 | 2,474 | 11.4 | 62 | 12.1 | 251 | 9.7 |
| Total number of patients in the subset | 4,417 | 100.0 | 21,777 | 100.0 | 511 | 100.0 | 2,579 | 100.0 |
| Number of men excluded for missing data on non-HDL | 1,297 | - | 6,668 | - | 181 | - | 881 | - |
| **HDL reduced to 1.1 mmol/L or decreased 10% ^4^** |  |  |  |  |  |  |  |  |
| No | 3,305 | 74.8 | 14,757 | 66.9 | 400 | 78.0 | 1,842 | 70.9 |
| Yes | 1,112 | 25.2 | 7,288 | 33.1 | 113 | 22.0 | 756 | 29.1 |
| Total number of patients in the subset | 4,417 | 100.0 | 22,045 | 100.0 | 513 | 100.0 | 2,598 | 100.0 |
| Number of men excluded for missing data on HDL | 1,297 | - | 6,400 | - | 179 | - | 862 | - |
| **nonHDL: HDL increased 20% ^5^** |  |  |  |  |  |  |  |  |
| No | 3,239 | 73.3 | 15,274 | 69.3 | 378 | 74.0 | 1,862 | 72.0 |
| Yes | 1,178 | 26.7 | 6,771 | 30.7 | 133 | 26.0 | 724 | 28.0 |
| Total number of patients in the subset | 4,417 | 100.0 | 22,045 | 100.0 | 511 | 100.0 | 2,586 | 100.0 |
| Number of men excluded for missing data on nonHDL:HDL | 1,297 | - | 6,400 | - | 181 | - | 874 | - |
| **Lipid-lowering therapy changes ^6^** |  |  |  |  |  |  |  |  |
| No | 4,493 | 80.2 | 22,412 | 90.2 | 594 | 87.5 | 2,788 | 82.4 |
| Yes | 1,112 | 19.8 | 5,521 | 19.8 | 85 | 12.5 | 597 | 17.6 |
| Total number of patients in the subset | 5,605 | 100.0 | 27,933 | 100.0 | 679 | 100.0 | 3,385 | 100.0 |
| Number of men excluded for missing data on lipid-lowering therapy | 109 | - | 512 | - | 13 | - | 75 | - |

^1^ We excluded those men without data on LDL at baseline in NDR register.

^2^ We excluded those men without data on TG at baseline in NDR register.

^3^ We excluded those men without data on total cholesterol and HDL at baseline in NDR register.

^4^ We excluded those men without data on HDL at baseline in NDR register.

^5^ We excluded those men without data on total cholesterol and HDL at baseline.

^6^ We excluded those men without data on use of stains or men with Ezetimibe at baseline.

*Abbreviations: PCa=prostate cancer; GnRH= Gonadotropin-releasing hormone agonists; LDL= low-density lipoprotein cholesterol; HDL= high-density lipoprotein cholesterol; nonHDL= non-high-density lipoprotein cholesterol; TG= triglyceride; NDR= national diabetes register.*
