# Supplementary figures and images for: Association between atherogenic lipids and GnRH agonists for prostate cancer in men with T2DM: a nationwide, population-based cohort study in Sweden
Source: Br J Cancer. 2022 Dec 15;128(5):814–24. doi: 10.1038/s41416-022-02091-z (PMC9977763; doi:10.1038/s41416-022-02091-z)

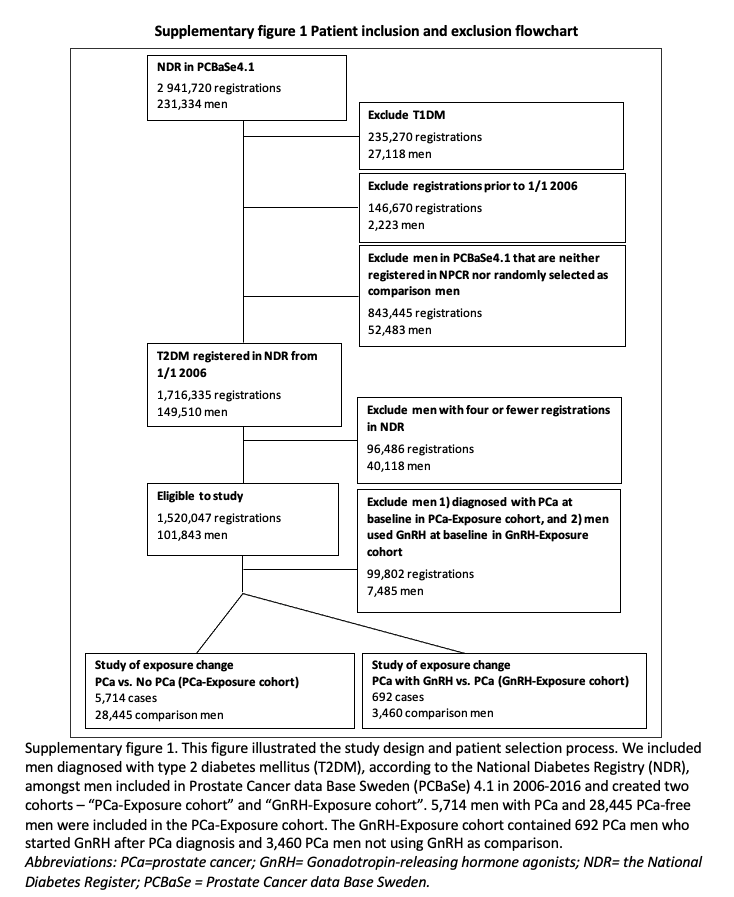

Supplement: Supplementary file 3 — Supplementary figure 1. Patient inclusion and exclusion flowchart [file 41416_2022_2091_MOESM3_ESM.png]

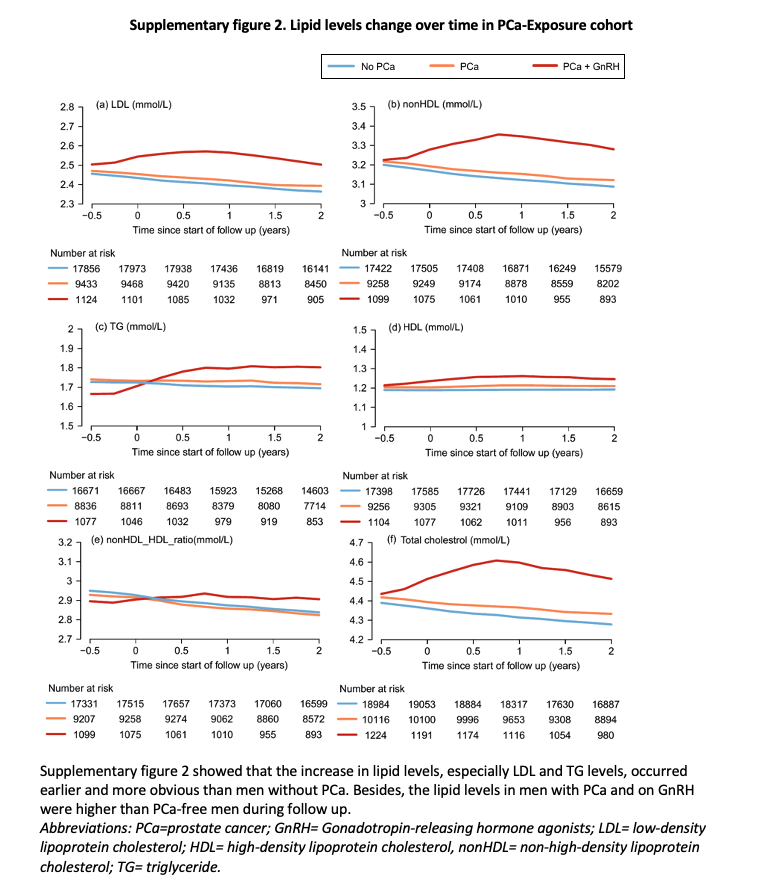

Supplement: Supplementary file 4 — Supplementary figure 2. Lipid levels change over time in PCa-Exposure cohort [file 41416_2022_2091_MOESM4_ESM.png]

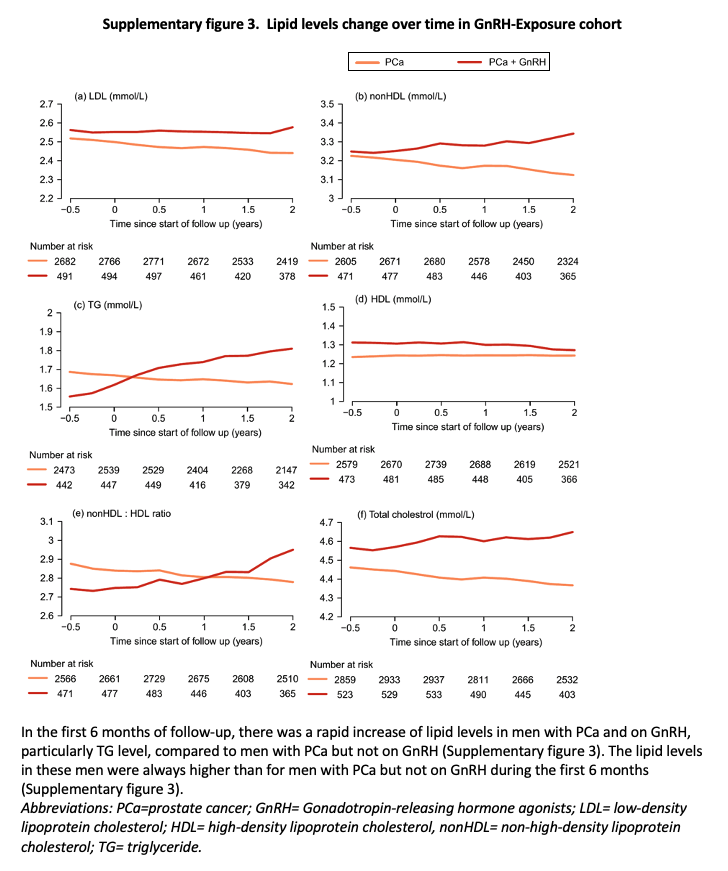

Supplement: Supplementary file 5 — Supplementary figure 3. Lipid levels change over time in GnRH-Exposure cohort [file 41416_2022_2091_MOESM5_ESM.png]
